# Young people’s proposals for tackling everyday challenges in order to improve mental health: a qualitative comparison study based on different socioeconomic neighborhoods

**DOI:** 10.1186/s12889-024-21147-8

**Published:** 2025-01-08

**Authors:** Helena Gard, Karin Ingvarsdotter, Gabriella Elisabeth Isma, Karin Enskär, Elisabeth Mangrio

**Affiliations:** 1 Department of Care Science, Faculty of Health and Society, Malmö University, Malmö, Sweden; 2 Department of Social Work, Faculty of Health and Society, Malmö University, Malmö, Sweden; 3 Department of Women’s and Children’s Health, Faculty of Medicine, Uppsala University, Uppsala, Sweden

**Keywords:** Everyday challenges, Mental health promotion, Qualitative comparison study, Socioeconomic inequities, Youth mental health

## Abstract

**Background:**

Everyday challenges and stress negatively affect young people’s mental health. Socioeconomic status (SES) is associated with different stressors and different stress-coping mechanisms. Many interventions target youth mental health, but few consider socioeconomic differences in the planning, implementation, or evaluation. In a Swedish context socioeconomic status is related with migration experience. The aim of the study was to explore proposals for tackling everyday challenges among young people from different socioeconomic neighborhoods.

**Methods:**

Eight focus groups, with participants between 13 and 15 years old from eight schools, were conducted in the south of Sweden. The participants discussed proposals for tackling everyday challenges. Using comparative thematic analysis, the focus group transcripts were divided into two groups, based on the socioeconomic status of the school’s neighborhood, and analyzed comparatively. Most of the participants in low SES neighborhoods had foreign background and most of the participants in high SES neighborhoods were Swedish born.

**Results:**

The analysis resulted in four shared themes between the two SES groups*: society is responsible, school is responsible, parents are responsible*, and *I am responsible*. The differences and similarities between the two groups are presented in sub-themes. Many of the proposals were similar between the two SES groups, but with different underlying issues and examples.

**Conclusion:**

Both groups proposed that adults must listen more to young people in order to improve the everyday challenges young people face. However, the low SES group in general expressed both more frustration and more agency, compared to the high SES group. This could be important to consider when planning school-based mental health promotion in different socioeconomic neighborhoods.

## Introduction

The effect of socioeconomic status (SES) on mental health and stress is well known, in both adults and young people [13, 36]. SES seems to affect stress among youth regardless of whether they live in families with low SES [13] or high SES [36]. Increased levels of stress were, for instance, found to be associated with parental criticism and psychosomatic symptoms for girls from high SES families [36]. In previous studies, young people themselves have argued that many of the symptoms interpreted as mental ill-health should rather be considered an effect of the everyday challenges experienced as a young person [35]. Everyday challenges identified by young people as stressful include norms and expectations related to race, gender, and socioeconomic status [11]. Therefore, there is a need to further explore proposals for tackling everyday challenges among young people from different socioeconomic neighborhoods, which was the aim of the present study.

The literature on differences in stress-coping strategies based on SES is sparse and differs between different measures of socioeconomic status. A Danish study examined the association between SES and active vs avoidant coping strategies, with active coping generally considered a more effective strategy. They found that there was a significant association between higher parental education and more active coping strategies among girls but not among boys [13]. Chen and Miller [7] similarly argue that active coping strategies for handling adversities are helpful for individuals from high SES-settings with access to resources but might actually have a negative effect on individuals from low SES-settings, aiming to actively cope with uncontrollable adversities. However, taking a different approach, involving accepting and adapting to the situation, could have a positive effect on resilience to negative stress-related outcomes among individuals from low SES-settings [7]. Hostinar and Miller [15] also identified protective factors that could increase resilience towards ill-health for people experiencing economic hardship during childhood. These factors included positive and close relationships with family, other adults and friends, a strong neighborhood community, and a positive school environment with effective classroom management and positive teacher expectations [15].

Many initiatives are being taken to reduce youth mental ill-health and stress. Existing school-based mental health promotion programs show some effect on mental wellbeing and suicide prevention. However, few are evaluated considering social factors such as SES, race, or gender [31] making it difficult to assess the effect of the programs in reducing mental health inequities. Sanchez et al. [25] also found that few intervention studies on universal school-based health promotion evaluated the effect of the intervention on socioeconomic equality, and that equality was rarely discussed in the studies. When it was addressed, it was primarily from the assumption that universal programs ensured a universal effect. The studies that did measure the effect on socioeconomic inequality were inconclusive in regard to the effect on inequality, in that universal interventions could have a positive, neutral, or negative effect on socioeconomic equality [25]. Socioeconomic segregation is a global issue, but it has increased in Sweden since the 1990s for those with the lowest and those with the highest incomes. These two groups live more segregated compared to the rest of the Swedish population [28]. People with foreign background are overrepresented in low SES neighborhoods and people with Swedish background are overrepresented in high SES neighborhoods [27, 33].

The current study was conducted within a larger research project called Authentic Life Intervention Challenges Everyday (ALICE). The purpose of the project was to develop and test a school-based intervention promoting mental health with a focus on young people’s everyday challenges. The project was conducted with schools in areas with different SES in southern Sweden and was conducted together with young people. In a previous study within the project young people related everyday challenges to race, gender, and SES, with SES is standing out as perceived to impact the everyday challenges young people face [11]. There is substantial knowledge about socioeconomic inequities in mental health and everyday stress, but there seems to be a lack of understanding regarding how this knowledge could be used in mental health promotion. To further understand how socioeconomic inequities could be handled in mental health promotion it is important to explore young people’s own understandings of what it takes to reduce everyday stress and challenges, considering the socioeconomic perspective. The aim of this study was to explore proposals for tackling everyday challenges among young people from different socioeconomic neighborhoods.

## Methods

### Study design

A qualitative comparison design, using focus groups at schools in different neighborhoods with both high and low SES, was chosen for this study. The purpose of carrying out a qualitative comparison analysis was to compare experiences and to deepen understandings of how the phenomenon can vary between groups [21]. Lindsay [21] describes how using comparison groups in qualitative research can contribute to understanding how differences between groups are manifested and constructed. It can also be useful to showcase biases regarding people from marginalized groups [21, 22].

The study has been approved by the Swedish Ethical Review Authority. Participants were informed about the purpose of the ALICE-project, what participation would entail, and about their right to quit study participation at any time. Furthermore, participants were informed about the study in writing and have signed written informed consent forms. For participants under the age of 15, a legal guardian has also signed the informed consent, in accordance with Swedish regulations.

### Study setting

The Swedish school system has ten years of compulsory schooling, grade 0–9 and grades are given from grade 6. During the 1990s the school system underwent several reforms, later criticized for contributing towards both socioeconomic and ethnic school segregation [2].

SES is strongly related to foreign background in a Swedish context. Foreign background is defined by either being born outside of Sweden or being born in Sweden and having two parents born outside of Sweden. In total 25% of the Swedish population has foreign background, compared to 74% in neighborhoods with low SES, in Swedish often referred to as vulnerable neighborhoods. Among young people less than 20% in vulnerable neighborhoods have Swedish background. Apart from Sweden, the most common birth countries for people living in vulnerable neighborhoods are Iraq, Syria, and Somalia [27]. In areas with the highest socioeconomic status are 89% of the inhabitants born in Sweden [33].

The young migrant group is diverse in regard to parents’ educational background in their countries of origin as well as labor market position and this differs between different nationalities. There are also differences in experiences of discrimination and racism between different migrant groups, with Somali migrants being particularly targeted [3].

Considering the study setting we understand SES through an intersectional perspective in this study. SES is not only directly related to income or educational level but also to other marginalizations acting as determinants for SES, for instance relating to race and ethnicity but also restrictive migration policies and inequalities [2, 18].

### Data collection

Multistage focus group discussions were chosen as the method of data collection as it is a good method to engage participants and explore understandings of a phenomenon on a group level. In focus groups data is generated from the interaction between individuals, rather than from individuals [1, 16], which was suitable for this study as school-based mental health promotion is often provided at group level. Each focus group met on three occasions, continuing their discussions from previous sessions each time. It was important for the study to create space for the voices of participants, rather than merely replies to the researchers’ questions. By meeting the participants several times, it gave them the opportunity to reflect and develop their discussions throughout the process [16].

Schools teaching the grades 7–9 in the southernmost part of Sweden were invited to participate in the larger ALICE-project. Seven schools participated in the original data collection in 2020 but for the present study one more school was recruited in order to increase socioeconomic variance, and those focus group discussions were conducted in 2022. Schools were invited using convenience sampling, using connections members of the research teams had from previous work. One member of the school staff at each participating school acted as a gatekeeper, inviting individual students to participate in the focus group at their school. Students who had an interest in everyday challenges, spoke Swedish, and did not have severe mental ill-health were asked to participate. The reason for excluding those with severe mental ill-health was to protect the privacy of the individual i.e., lower the risk of participants sharing about their own health instead of taking part in a more general discussion about everyday challenges. None of the participants dropped out of the study, however there were some variances in participants throughout the different sessions, due to illness.

In total, eight groups participated in three focus group discussion sessions per group. In total 73 young people participated in the study and between four and 12 participants took part in each session. Each session lasted about an hour and a half. Three of the groups were in neighborhoods with socioeconomic challenges, and five of the groups were in neighborhoods with good socioeconomic conditions (see Table 1). The focus groups met at the participants’ school during school hours. Two researchers facilitated each focus group, one acting as moderator and the other as note-taker, only the two researchers and the participants were present in the room. The first, second, third, and fourth authors conducted the focus groups. The researchers facilitating the focus groups all had previous experience in working with teenagers in health care and health promotion but had no previous relations to the participants in the study.

**Table 1:** The participating focus groups

**Low SES group**		
School L1	Area with large socioeconomic challenges (type 1)	10 participants
School L2	Area with socioeconomic challenges (type 2)	10 participants
School L3	Area with large socioeconomic challenges (type 1)	8 participants
**High SES group**		
School H1	Area with good socioeconomic conditions (type 4)	8 participants
School H2	Area with good socioeconomic conditions (type 4)	4 participants
School H3	Area with very good socioeconomic conditions (type 5)	12 participants
School H4	Area with good socioeconomic conditions (type 4)	10 participants
School H5	Area with good socioeconomic conditions (type 4)	11 participants

In the first session, the participants were asked to identify everyday challenges, these included school problems, body issues, relationships, and inequities [11, 14]. In the second session, they were asked to discuss possible solutions for everyday challenges, and in the third session they were asked to discuss ideas for a mental health promoting intervention targeting everyday challenges. For this study, only the second and third sessions were included.

At the beginning of the second and the third discussion session, the moderator summarized the previous session, asking if the participants recognized themselves in the summary. The sessions then started with an open question; for the second session this was: “What could be done about these everyday challenges that you have identified?”, and for the third session: “How could an intervention targeting these everyday challenges be constructed?”. In practice, the discussions in the second and third sessions were similar and were analyzed together for this study.

### Study population

The participating schools were divided into two groups based on the SES of the area, using an index from the Swedish government. The index is a five-grade scale with 1 being an area type with large socioeconomic challenges and 5 being an area type with very good socioeconomic conditions [32]. Three of participating schools were in areas of type 1 or 2, and five of them were in areas of type 4 or 5, whereas no schools were in areas of type 3 (see Table 1). The intention was to recruit more schools in area type 1 or 2, but this proved difficult, making the group sizes uneven. This index was used to divide the participating schools on a group level based on geographical location.

The low SES group consisted of young people at three schools in southern Sweden. The 28 participants were of different genders, between 13 and 15 years old and most of the participants had either their own or parental migration experience. The neighborhoods where the schools were located were characterized by socioeconomical challenges, defined as relatively high levels of low-income households and low education levels, as well as high levels of unemployment [28].

The high SES group consisted of young people at five schools in southern Sweden. The 45 participants were of different genders, between 13 and 15 years old and few of the participants had migration experience. The neighborhoods where the schools were located were characterized by good socioeconomic conditions defined as relative low levels of low-income households and low education levels, as well as low levels of unemployment [28].

### Data analysis

The data analysis was overall inspired by comparative qualitative analyses [9, 24] and thematic analysis according to Braun and Clarke [4] was used. A thematic analysis approach for coding and sorting the data in the analysis was chosen, as it is a flexible method to explore patterns in the data [4] and this fits well with the comparative design. Braun and Clarke [5] describe how themes created should be based on shared meanings, rather than concepts, and how they should tell a story about the data.

**Fig. 1:**
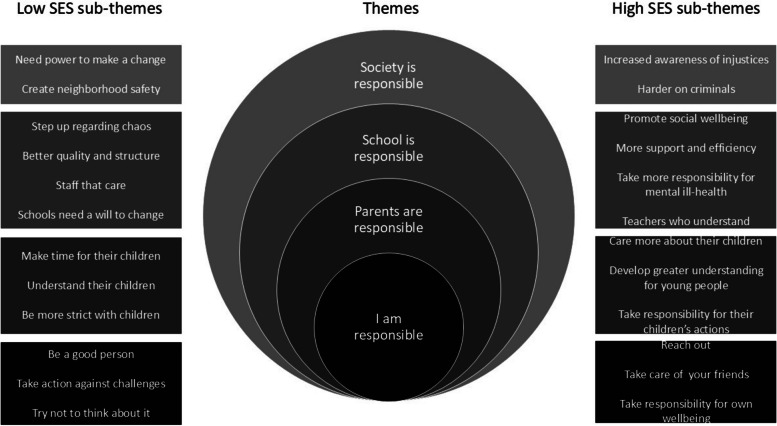
Themes and sub-themes

The transcripts from the eight focus groups were divided into two groups based on SES. Lindsay [21] identifies different approaches to data analysis in comparative qualitative research, coding all the data together inductively and then making comparisons between the groups, or identifying commonalities between the groups first before coding deductively. For this study, the latter approach was chosen and themes shared between the two groups were identified initially by the first author when reading through each transcript (see Fig. 1). This reading included searching for meanings on both a semantic and a latent level, in accordance with Braun and Clarke [4]. In the next step of the analysis the transcripts were coded in what they had to say about each shared theme. These codes were then organized and reorganized into sub-themes, based on their interpreted meaning, separately for each SES group. After the creation of the sub-themes for each group, the sub-themes were juxtaposed for comparison. In the result section, the sub-themes with the differences and similarities between the two SES groups are presented under the shared themes. The software NVivo 14 [23] was used for coding and organizing the data. The first author did the coding and organizing into preliminary sub-themes, however the process included recurring discussions with the co-authors to promote reflexivity throughout the process [4]. The codes, sub-themes and shared themes were developed throughout the process of discussions back and forth between all co-authors to reach consensus in the analysis.

## Results

The first part of the analysis resulted in four shared themes concerning which actor was responsible for implementing the proposals to tackle everyday challenges: society is responsible, school is responsible, parents are responsible, and I am responsible. The proposals were expressed both as needs for change and as concrete suggestions. Further analysis in the two groups resulted in several sub-themes under each theme (see Fig. 1).

### Society is responsible

The need for change and an ambition to find solutions to young people’s everyday challenges on a societal level, were present in both groups. Problems in school or in families were discussed as being connected to society, making society co-responsible for problems related to family poverty or chaos in schools. There is, participants asserted, a need for fast changes in society and it was seen as a waste of time that the problems are not addressed by those with power. The participants in both groups raised the need for societal change to create a more just society, stressing that it is important to learn and gain knowledge about the surrounding society, both locally and globally. However, in the high SES group, the need for* increased awareness of injustices* was discussed as mainly being located somewhere else, in other parts of the world. It was seen as important to feel solidarity with others and to want to make a change in other countries.

“I just think it’s important, considering the world we live in right now, that young people get an early view, sort of. And I know, we have the internet and find out fast about things, but to really get to know how serious it is and watch the news at school, or I don’t know, something…”.

School H1

Both groups discussed the need for society to address criminality. The high SES group again mainly discussed this as something that needs to be done somewhere else, while the low SES group referred to their own neighborhoods and experiences. The participants in the low SES group discussed the need to *create neighborhood safety,* relating this to safety regarding both criminality and poverty. There was optimism in the low SES group, that change could happen and actually is happening for the better, but at the same time there was an understanding that change is very difficult. A solution discussed was for more, as they described them, Swedish people to move to their neighborhoods, which they described as mostly inhabited by immigrants. They argued that Swedish people are more calm because they have not experienced war, and this would have a positive effect on the neighborhood.

“What I’m trying to say is if Swedish people come here, to this neighborhood, I think it would get a lot more calm. Because you see how Swedish people are, they are calm.”

School L3

This suggestion was also questioned, with arguments stating that the lack of Swedish people was a good part of the neighborhood but also saying that Swedish people would not want to live in a neighborhood like theirs. The low SES group discussed that it *takes power to make a change* in society. Those in power include politicians or Swedish people, as opposed to immigrants. The participants expressed that they themselves have little power, but that they could raise issues with those who have more power. They suggested that an intervention could entail students going to politicians and inviting the prime minister to the school.

“So he can meet the kids and the worst school in Sweden.”

School L1

Participants in the high SES group argued that society needs to be *harder on criminals* and discussed that harsher consequences in general could act as a deterrent. Others also stressed the importance of society not glorifying a criminal lifestyle.

“Society should also change so that you don’t glorify criminality, that it’s seen as cool to, for example, murder someone or be part of a gang and stuff.”

School H4

The participants discussed punishments as a deterrent but also emphasized the need for harsher punishment related to crime, bullying, and drugs, to show young people that these behaviors are unacceptable.

### School is responsible

Both groups expressed the great need for changes in their schools as well as many suggestions on how schools could take responsibility for reducing stress and everyday challenges for young people. The low SES group described their school environment as chaotic, claiming that to address this the schools need to *step up regarding chaos.* Participants discussed that schools need to act against, what they called, the chaos caused by students’ behavior. There should be harsher punishments and consequences for students who disturb others in the classroom.

“If someone is being disturbing, maybe one or two students. If you tell them to stop and they don’t stop, take them out of class. Because they don’t understand that they are disturbing everyone else. Who maybe want to concentrate on what they are doing and finishing some work or something.”

School L1

Other consequences included calling the parents or kicking students out of the lesson. But schools should also be more lenient regarding minor things, such as getting to have your phone in class and not being reported absent if you are only a few minutes late to class.

The low SES group also discussed the need to do activities with the class outside of school, such as field trips to nature sites or to a museum, as a possible solution for improving the school environment. Others argued that this could never be done at their school, as their classmates were too disruptive and did not deserve to go on field trips.

To get to do activities outside of school was described by both groups as important for the feeling of togetherness and to get a break from the everyday stress caused by school-related pressure. The high SES group also stressed how important it is for schools to *promote social wellbeing* of the students. One proposal discussed, similarly to the low SES group, was arranging organized, social activities during school hours. Activities suggested involved going on field trips to the forest or visiting museums. This was considered especially important for classes who had conflicts.

Because you have to spend time together in a different way and get a better sense of unity.

School H2

Participants thought it was a good idea for the school to force students to interact with each other, but others argued that they could not befriend someone just because the teacher told them to.

The high SES group also expressed the need for schools to step up their consequences for misbehaving students in order to promote student wellbeing. Harsher consequences for bullying were uppermost in this discussion. Adults at the school need to be more observant of the students’ interactions and step in when they see bullying. Stepping in could involve talking to the bullying student as well as their parents. Punishments, such as detention, were suggested. But participants also expressed awareness of the fact that bullying is not an easy problem to solve and that it could be difficult for the teacher to discipline students.

“They aren’t allowed to do a whole lot. If they tell someone, someone who disturbs the lesson, that they have to leave, they can’t do anything other than telling them ‘you have to leave.’ If they don’t leave, they can’t do anything. They are not allowed to touch the student or anything, they don’t get to remove the student or drag the student out, or anything. They can’t, for if they do, they will be reported.”

School H1

Both groups requested schools to be better. The high SES group discussed the need for *more support and efficiency* in school, which included being involved in decisions that affect them, such as rules for cell phones or school hours. Participants felt that the school does not listen to them and that they have little power over their situation. The high SES group also discussed that the requirements for getting a high grade are too hard to reach and that there is a need for more efficient school days in order to improve student performances. This could involve teachers planning together, to even out the student workload. Other suggestions involved scheduling, with shorter breaks and thereby shorter days, or longer breaks so that students would have time to do homework during breaks. They also requested more support from teachers, when planning their homework or when studying for exams. Support could, furthermore, be provided by having more teachers, or by teachers having more time to help students during class. Less or no homework was also considered preferable. Moreover, participants were aware that their teachers do not have a good working environment and that teachers should be able to spend their time teaching instead of having other duties.

“So the teachers have to stand there to let people in to the cafeteria. And that is taken from the time they have to teach.”

School H1

The low SES group also identified several factors necessary for *better quality and structure* in the schools. One change they requested was better school lunches and access to snacks, such as fruits, during school hours. Some discussed the need for a cafeteria where students could buy cheap snacks. Some schools in the group had had a cafeteria before, but it had closed; they discussed it as unfair that other schools have access to a cafeteria while they do not. School lunches were described as being poorly cooked and often consisting of dishes that are not preferred by young people, schools were suggested to serve meals from different cultures and not just Swedish meatballs and potatoes. Better scheduling was also discussed as important for reducing everyday stress in the low SES group. They discussed the need for teachers to talk to each other and plan better.

Participant 1: And not have tests every week and reports every week.

Participant 2: The teachers, in their teams, they should agree on which tests they are having, so that we don’t have all the tests the same week.

Participant 3: Better planning.

Participant 1: One week we had four tests.

School L1

Other planning suggestions included starting school later in the morning, ending school earlier in the afternoon, longer breaks, and more breaks. Participants acknowledged, similarly to the high SES group, that it would be difficult to meet everyone’s wishes regarding school hours, but that something has to be done to create a more reasonable schedule with decent breaks. Longer school days could be okay if it meant that they would not have homework, as finishing schoolwork in school was considered preferable to doing it at home.

The high SES group discussed that the schools should *take more responsibility for mental ill-health* by providing knowledge about mental health as well as offering support to those with mental ill-health. Informing about mental health was discussed as something that normalizes mental ill-health, making it clear that it is okay to feel bad. Participants mentioned the importance of teaching students about eating disorders, self-harm, and suicide, also discussing that it is important to learn about mental health from a young age and suggesting that older students could talk to younger students about this. Furthermore, the school could take responsibility in teaching parents about mental health, and participants suggested that this could be done at the regular parent-teacher meetings.

Interviewer (I): “You could have mental ill-health as a theme in different ways?”.

Participant (P) 1: “Yeah, the counselor or someone else could come and talk to the parents, [saying that] ‘yes, this is in fact the way it is’.”

P2: “Yeah, it’s so common that there’s nothing strange about it.”

School H2

The low SES group also discussed the role of the counselors, in relation to the need for school *staff that care* about the students*.* Participants expressed a distrust in the school, making some health promotion initiatives questionable. They did not trust the confidentiality of counselors or of anonymous surveys, expressing that they could not be honest with their answers due to lack of trust or because they thought schools should not ask questions that were “haram”.

There is, participants said, a need for school staff, including teachers, teacher assistants, and counselors, to see the individual student and not only give generic advice, thereby minimizing the student’s problems. Some expressed the need for younger people among school staff, as younger people understand them better than older people, similarly to the reasoning in the high SES group. Participants felt that many teachers should show the students more respect and stop comparing them, by identifying them as immigrants, to Swedish students.

“How do you want us to respect her, the teacher, if she doesn’t respect us back? How she treats us, how she treats us with disrespect, well she puts us down, she barely knows our answers. [If] we haven’t managed something, she judges us, saying that we won’t make it. Why should we respect her when she does that?”.

School L1

The high SES group, similarly, discussed the need of *teachers who understand* the students, for instance by not forcing students to make presentations in front of the class if they do not feel comfortable with that, or by giving them the possibility to take a re-exam. Teachers also, participants claimed, need to keep up with the situation of young people today, concerning, for example, technology or mental health. They should, moreover, treat the students well and with respect, to show that they care.

“That you get more support from teachers, rather than pressure. If you’re feeling bad during class and you can’t work or something, usually you get more pressure that you have to work, instead of asking what has happened and stuff.”

School H5

In general, teachers should be more supportive than pressuring; they should correct mistakes but do it in a friendly way. Talking more about mental health could increase the trust in the teachers.

Among the participants in the low SES group there was a belief that their *schools need a will to change* and that students could influence this process. They argued that it might be too late for positive changes and that the school does not listen to, or care about, the opinions of the students. Others suggested bypassing the principal and talking to those higher up in the hierarchy, such as the school management in the municipality, responsible politicians, or the Swedish National Agency for Education. Participants stressed that the ideas and opinions of the students are necessary in creating positive change and argued that politicians had to listen to them.

P1: “Because we are the future, or else we will remove the whole school management, the whole government. Our own political party!”.

P2: “Yeah, what is the school without students?”.

P3: “Because the goal of the school is that you should like it here and learn something. But it’s not happening.”

School L3

According to participants, schools also need to be more transparent with their actions for change. They expressed that they are being listened to and that they are being asked about their opinions on different matters, but then nothing happens.

### Parents are responsible

Both groups expressed needs and solutions related to parents needing to take more responsibility for their children’s actions and well-being. The high SES group discussed that parents have to *care more about their children.* Parents need to be more supportive of their children in handling their everyday challenges, related to school, social media, and mental health. The participants emphasized that parents should be good role models and teach their children how to handle struggles in life. One suggestion presented was that parents need to engage more with the school in order for the parents to take school more seriously and be able to be supportive.

“… parents should be more engaged in the children’s life. Because parents often don’t know how the children are doing or what they are doing.”

School H3

Another responsibility of the parents, according to the high SES group, is to help their children stay motivated, regarding, for example, achievements in school and sports. It was seen as important for the parents to give positive and negative feedback so that their child could develop.

A similar discussion was evident in the low SES group, but the need expressed by the participants in this group was that parents should *make time for their children*. Parents should make time by looking up from their phones.

“Even if they have time, they have to check Facebook, Instagram or something first.”

School L1

They should be there when their children need them, believe in their children and trivialize their children’s problems or gossip about their children to relatives.

Both groups discussed the need for improvement in the way their parents understood them. The low SES group argued that society has changed since their parents were young and that parents need to *understand their children* and their situation today.

“If we talk about going out he always says ‘in my days girls were like this and this’. Well, it is 2022, it’s not your days anymore.”

School L2

It was seen as difficult to change the attitude of parents who are old-fashioned, and participants discussed that maybe it is better not to tell parents everything, or not be at home too much, since the parents will not understand their children anyway. Participants in the low SES group also discussed that there is a need for their parents to learn about the Swedish school system, but at the same time participants understood that it might be difficult when their parents do not speak Swedish.

The high SES group discussed that adults in general, but especially parents, need to *develop greater understanding for young people* and that it is their responsibility to grow this understanding. Parents also need to learn more about the mental health and self-confidence of children, in order to develop a more relaxed view of mental ill-health, as the participants considered mental ill-health a natural part of everyday challenges for young people. The participants in the high SES group, similarly to the low SES group, discussed that parents have to understand that society has changed.

“I think you should raise with parents, like teach the parents. Our parents, like thirty or forty years ago, maybe they didn’t have these problems, like in the same way we have with mental ill-health today.”

School H2

One suggestion was to film interviews with young people to show the parents, so that parents could understand that their child is not alone in having the experiences of mental ill-health in question.

Further, the high SES group argued that parents should *take responsibility for their children’s actions*. Participants emphasized the responsibility of the parents to teach their child how to behave towards others, and that bullying, and prejudice, is wrong.

“Then the parents have to teach that. That you are often more than what you look like. You have to learn respect.”

School H4

Parents also need to realize that their children might behave differently, that is, worse, when they are not at home.

Participants in the low SES group also discussed parental responsibility and argued that parents should *be more strict with children*. This was discussed as necessary in order to reduce chaos during school days and improve the school climate for other students. Parents should punish their children so that they can learn and change their behavior, and the need for consequences for bad behavior was emphasized. Even if strict parents could be annoying, participants saw it as better than the opposite.

P1:”Well, actually, some [families], when you, like, look at it. Arab families, they are a bit annoying but it’s still fun to have one that’s not Swedish.

P2: “You joke a lot about it…”

P1: “It’s more fun to have an Arab family that is strict, than a Swedish one that is, like, very soft and stuff.”

School L3

Both groups emphasized the need for parents to take more responsibility for their children’s actions and be more understanding towards their children. The participants in the low SES group stressed that their own parents were strict, which they in general saw as something positive, and that other parents also need to be more strict. The high SES group stressed the need for parents, or adults, to understand the general situation for young people today, while the low SES group discussed the need for parents to commit to understanding their individual children better.

### I am responsible

Apart from what others should do, there are also things oneself could do in order to change everyday life for the better, as discussed in both groups. This involves taking care of oneself but also making an impact for other people. The high SES group raised that one thing a person could do if feeling bad is to* reach out* and talk to someone else, such as a friend, an anonymous help line, a mental health professional, or a parent. The benefit of talking to a professional is, the participants said, that they have professional secrecy. Sometimes the participants could speak to their parents, but not if they were the ones causing stress.

“… you could talk to an adult that you can get help from, if you can’t talk to your parents. And you could also be pressured by your parents, if there’s a test or something during the week that you haven’t studied for, or something.”

School H5

The high SES group also discussed what one could do if one feels bad due to a conflict with someone, saying that it is important to express to their partner or friend how they feel. Moreover, it could be easier to write, rather than talk, about one’s feelings.

Another way to take one’s own responsibility discussed by the high SES group was to *take care of your friends*. It could be difficult to know what to do when a friend is not feeling well, they said, but it was still seen as important to take responsibility for one’s actions towards friends. Friends should have each other’s backs and help each other. But at the same time, it was seen as important to know one’s limitations.

“But then you have learnt that you can’t affect everyone, how they are against others, unfortunately. So that’s just something you have to learn.”

School H1

Participants also discussed the importance of letting go of bad friends, and of finding new ones who treat you better.

Similar topics were discussed by the low SES group too, but in the context of to *be a good person* to others in general*,* to not bully or call others bad names.

“First of all to not say something inappropriate, to not say bad words to each other, because it makes someone feel worse and that’s just unnecessary.”

School L2

This included helping peers who have gotten into trouble and apologizing if one has misbehaved. Participants discussed that it could be frustrating to try to help a friend who does not want to receive help, suggesting that then it might not be worth it. They acknowledged that it is not always possible to help a friend, but that you could try to talk with them to find a solution.

Participants in the low SES group discussed the importance of having their own strategies in order to *take action against challenges*. Participants suggested that you could talk to someone if you feel bad, such as a teacher or a friend. But this could also be challenging, as it is not always possible to trust others, and then it could be better to keep things to oneself rather than risk a spread of rumors. When it came to schoolwork, it was deemed important to have self-discipline and not only blame the teacher, but also take responsibility for one’s own work. It could also help to listen to motivational speeches on YouTube, pray, and work to improve one’s self-esteem. Strategies involved planning schoolwork and talking with teachers about adaptations.

“If you’re shy or something, you don’t dare speak in front of the whole class. Then you could tell your teacher that will understand why you don’t dare and… If it’s, like, a presentation or something, you could do it in a smaller group. With people, like, you feel comfortable talking in front of.”

School L2

Everyone, the participants said, has a responsibility to act against bad things, such as other students disrupting lessons. You could take responsibility by not encouraging students who misbehave, by ignoring them, or by talking to the principal.

The high SES group discussed to *take responsibility for own wellbeing*. Something that a person could do, regarding school and sports, is to become more structured by planning and working in a more goal-oriented manner. Participants also suggested working on improving study techniques or hiring help for homework. Another way to deal with stress could be to distract oneself by doing something fun, such as listening to music, reading, going for a walk, or hanging out with friends. Participants discussed that it is also important to learn to stand up for oneself. This was discussed as being easier said than done, but it could entail trying not to get stressed over grades, blocking people who make you feel bad on social media, believing in oneself, and not comparing oneself to others.

“Yeah, maybe that’s mostly where you compare yourself, or maybe to people close to you. And then you should maybe focus on yourself and, like, promote yourself.”

School H5

The participants in the low SES group also discussed that, apart from taking action, one strategy could be to ignore and escape one’s everyday challenges and *try not to think about it.* This could be done by hanging out with friends, working out, sleeping, or watching a show. It could also help to ignore people who do not act right, on social media and in school.

“Also, overthinking, we overthink way too much.”

School L3

Resorting to distractions, as suggested, could work for a while, but participants emphasized that it is also important to find solutions to the underlying problems.

## Discussion

The analysis showed both similarities and differences between the low and the high SES group in proposals for tackling everyday challenges, possibly reflecting similarities and differences in everyday conditions between the neighborhoods. During the focus groups and in the transcripts, the discussions at first appeared very different between low SES and high SES schools. But further on, in the analysis, when the sub-themes from each group were juxtaposed, and the meanings the participants attached to each shared theme were analyzed, instead of how they expressed the proposals, many similarities became clear. Similar proposals were primarily presented regarding school, for instance the need for better organization around school tasks, and regarding parents, for instance the need for parents to make time for and care about their children. The differences are in the nuances, but these nuances also speak of the differences in everyday life and in the challenges experienced; for instance, both groups discussed proposals relating to criminality, but living in a neighborhood with high criminality is different from considering criminality a problem somewhere else. Both groups made suggestions for solutions that involve young people themselves taking action, and they problematized that they are not always listened to, or taken seriously, by adults in their surroundings.

The low SES group was in general more pessimistic of the possibilities of change but at the same time more committed to contribute towards change, compared to the high SES-group. The high SES group saw needs for change but did not express as strong a commitment to contribute towards that change. The differences in commitment to change, or in being motivated to fight for change, could be related to social capital theory. Claridge [8] discusses different levels of social capital, from individual to group or community level. Group level social capital can be had in different groupings, for instance based on neighborhood, class, or race [8]. Even though we do not know the SES of the individual participants in this study, it could still be discussed under the concept of social capital, on a group level. The result of this study implies that the low SES group felt a stronger sense of community, or grouping, based on their neighborhood, compared to the high SES-group. This might, considering social capital theory, have increased a sense of social capital in the low SES-group, which could contribute to explain the fact that they expressed more agency and drive to make a change.

The low SES group also compared their community to other communities to a larger extent than the high SES group did. This included comparisons both with other schools considered better and with Swedish people, as many identified themselves as immigrants despite being born in Sweden, possibly reflecting the discourse of race and migration in Sweden. Most of the students at the low SES schools were immigrants or children of immigrants, while most of the students at the high SES schools lacked migration experience and were born in Sweden. Globally, as well as in Sweden, low socioeconomic status is often associated with other types of marginalization, such as belonging to a racial minority and having own or parental experiences of migration [28]. Considering the intersectional perspective is crucial when interpreting the results, as the similarities and differences between group cannot be said only to be associated with economic differences but also differences in migration experience, cultural background, personal experiences of restrictive migration policies, and experiences of racism [18].

The participants in the low SES group discussed that hearing that their school was the worst in Sweden could increase the motivation to be successful and prove those who claimed this wrong. Even though this coping strategy might be seen as something positive, it could possibly increase stress. For instance, Brody et al. [6] found that rural, black adolescents from low SES families in the United States with high scholarly and social competence, had low levels of adjustment problems, but high levels of psychological stress. This reflects the John Henryism theory that people who are experiencing severe economic and racial disparities and at the same time are expected to excel academically to “beat the odds” may seem to do well in a conventional sense, but eventually manifest signs of psychological stress [6, 17].

The results also highlight the need for changes at four different levels: society, school, parents, and self. This aligns with theories considering multi-level causations of health and illness, such as Eco-social theory [20] or the Rainbow Model [10]. Many mental health promotion programs target only one of these levels, often the individual level [29, 30], which could be argued to put the responsibility of youth mental health solely on young people themselves. Universal programs used in Sweden for preventing mental ill-health in teenagers, are mostly aimed at the individual level, such as Depression in Swedish Adolescents [12], Social and Emotional Training [19], and Youth Aware of Mental Health [34]. According to the governmental agency (2021), few municipalities in Sweden use universal programs for parental support. To the best of our knowledge, no intervention programs in Sweden aiming to prevent mental ill-health, or promote mental health, target the societal or school level. To address the needs and proposals suggested in this study, a multi-level approach would be needed, taking into account not only the individual level but also the parental, school, and societal level.

### Methodological considerations

The choice to make a comparative thematic analysis, rather than a thematic analysis on the material as a whole, contributed to showing variance between the different SES groups. Even though the method aims to find differences between groups, the analysis in this study also found many similarities between the two SES groups. Lindsay [22] argues that using comparison groups in qualitative research can be a way to address bias and highlight assumptions about the experiences of different groups of young people. It can, however, also be a challenging approach, as it is not very well explored methodologically in the literature [21]. The authors have tried to handle this by being clear in describing the process of the analysis to enhance the rigor of the study. The fifth author did not participate in the data collection and brought an outside perspective on the data in the analysis, which contributed to reflexivity in the process.

In the conducted focus groups within this project the overall topic has been everyday challenges. This has not been discussed as a sensitive topic within the project, but on the other hand we cannot know what can be sensitive to someone else [26]. An ethical concern relating to conducting focus groups with young people has been to create a safe space for the participants to share their views and experiences, connected to the social dynamic within the group. The participants attended the same school, and many knew each other. They were invited to participate in the focus groups by a member of the school staff who knew the students, but there is still no guarantee that the staff member in question knew about potential conflicts or bullying within the group. Sim and Waterfield [26] problematize the issue of confidentiality in focus groups. As a focus group moderator, it is hard to predict which topics will be discussed in a focus group and the discussion can take unexpected turns. Participants might not feel safe to disclose their views in the group, or they might experience not being able to share their views due to other participants dominating the discussion [26]. On one occasion, the participants explicitly expressed distrust in each other, and this was discussed with the participants, but they still wanted to continue. The moderator continued the discussion with extra caution, but the distrust expressed could have influenced what the participants felt comfortable sharing.

There are several limitations to this study. A limitation of the data collection could be the use of gatekeepers at participating schools to invite individual participants, in that the researchers renounced some control over the sampling process. This could have skewed the sample and, at schools described by the participants as filled with conflict, students considered well-behaved may have been favored when gatekeepers chose whom to invite. The authors also do not know how many students declined participation, or why, when asked by the gatekeeper and how the possible missing perspectives of the non-participants may have affected the results, which is another drawback. Other data collection methods apart from focus groups, by the use of triangulation, could have been used that might have captured more experiences and could have contributed towards compensating for this limitation.

Another limitation of this study is the uneven group sizes. It was more difficult to recruit schools in low SES areas than in high SES areas, the low SES area schools often referring to lack of time or poor school results when declining participation. This possibly reflects the everyday challenges described by participants in this study. The demographics of the two SES groups were expected in a Swedish setting, however it may make the results less transferable to countries with less migration or ethnic and racial diversity.

The individual SES of the participants was not collected, and it is possible that some of the students in the schools did not live in the neighborhood where the school was located. This division of groups based on neighborhood SES data, rather than individual SES data, could be a drawback, as there is no way of knowing the individual SES of participants. However, this study aimed to explore socioeconomic variance in tackling everyday challenges on a group level, in order to gain an understanding of how this knowledge could be used in school-based mental health promotion. The study could thus contribute to understanding acceptability of different proposals for change on school level, regardless of the SES of the participating individuals.

## Conclusion

The young people at schools in neighborhoods with low SES and in neighborhoods with high SES, shared many similarities regarding needs for change and practical proposals, but they expressed them and exemplified them in different ways. Both groups expressed a need for adults in families, schools, and society to listen to young people, suggesting how adults could listen. However, the low SES group expressed not only more frustration and hopelessness but also more agency and determination to make a change, compared to the high SES group, who expressed less frustration but also less determination to contribute towards positive change. Even if an everyday challenge and a proposed solution is similar, it is still important to consider the nuances of different lived experiences and to consider how this is expressed. It is difficult to attribute these similarities and differences to specific aspects of class, financial situation, religious beliefs, cultural background, migration experience or experiences of discrimination as this was not the scope of the current study and also being informed by intersectionality these factors are not possible to entangle. However, school-based mental health promotion is generally done at group level and the differences and similarities between the schools in different SES neighborhoods could still be important to consider when planning mental health promotion, even without knowing underlying mechanisms.

The results of this study indicate a need for a multi-level approach in order to address the needs and proposals of young people. Future interventions aiming to promote mental health and mental health equity could benefit from such a multi-level approach, taking into account both the different levels an intervention could target and consider structural determinants such as socioeconomic status, in order to promote mental health equity. The results also emphasize the importance of including young people themselves in the design of interventions.

The knowledge gained from this study could be important to consider in school-based health promotion, both in the differences and the similarities between different SES groups. Perhaps the content of an action could be similar between different socioeconomic neighborhoods, but the delivery of that action should differ and be contextualized. Adapting health promotion to reduce inequities is a balance, however, in considering differences without exaggerating them, or without othering those traditionally marginalized. Based on the participants’ experiences and proposals, it would be interesting to further explore discourses, attitudes, and experiences regarding socioeconomic inequities in mental health promotion, from the perspectives of policy and professionals as well as considering other structural determinants such as gender or race.

## Data Availability

The dataset analyzed during the current study is available from the corresponding author on reasonable request.

## Abbreviations

SES: Socioeconomic status
ALICE: Authentic Life Intervention Challenges Everyday
